# HER2, chromosome 17 polysomy and DNA ploidy status in breast cancer; a translational study

**DOI:** 10.1038/s41598-019-48212-2

**Published:** 2019-08-12

**Authors:** Altuna Halilovic, Dagmar I. Verweij, Annet Simons, Marian J. P. L. Stevens-Kroef, Susan Vermeulen, Janet Elsink, Bastiaan B. J. Tops, Irene Otte-Höller, Jeroen A. W. M. van der Laak, Carlijn van de Water, Oliver B. A. Boelens, Margrethe S. Schlooz-Vries, Jeroen R. Dijkstra, Iris D. Nagtegaal, Jolien Tol, Patricia H. J. van Cleef, Paul N. Span, Peter Bult

**Affiliations:** 1 Department of Pathology, Radboud university medical center (Radboudumc), Nijmegen, The Netherlands; 2 Department of Tumor Immunology, Radboud university medical center (Radboudumc), Nijmegen, The Netherlands; 3 Department of Human Genetics, Radboud university medical center (Radboudumc), Nijmegen, The Netherlands; 4 Department of Surgery, Hospital Pantein, Boxmeer, The Netherlands; 5 Department of Surgery, Radboud university medical center (Radboudumc), Nijmegen, The Netherlands; 6 Department of Medical Oncology, Radboud university medical center (Radboudumc), Nijmegen, The Netherlands; 7 Present Address: Department of Medical Oncology, Jeroen Bosch Hospital, ‘s-Hertogenbosch, The Netherlands; 8 Radiotherapy & OncoImmunology laboratory, Department of Radiation Oncology, Radboud university medical center (Radboudumc), Nijmegen, The Netherlands

**Keywords:** Breast cancer, Breast cancer

## Abstract

Breast cancer treatment depends on human epidermal growth factor receptor-2 (HER2) status, which is often determined using *dual* probe fluorescence *in situ* hybridisation (FISH). Hereby, also loss and gain of the centromere of chromosome 17 (CEP17) can be observed (*HER2* is located on chromosome 17). CEP17 gain can lead to difficulty in interpretation of HER2 status, since this might represent true polysomy. With this study we investigated whether isolated polysomy is present and how this effects HER2 status in six breast cancer cell lines and 97 breast cancer cases, using HER2 FISH and immunohistochemistry, DNA ploidy assessment and multiplex ligation dependent probe amplification. We observed no isolated polysomy of chromosome 17 in any cell line. However, FISH analysis did show CEP17 gain in five of six cell lines, which reflected gains of the whole chromosome in metaphase spreads and aneuploidy with gain of multiple chromosomes in all these cases. In patients’ samples, gain of CEP17 indeed correlated with aneuploidy of the tumour (91.1%; p < 0.001). Our results indicate that CEP17 gain is not due to isolated polysomy, but rather due to widespread aneuploidy with gain of multiple chromosomes. As aneuploidy is associated with poor clinical outcome, irrespective of tumour grade, this could improve future therapeutic decision making.

## Introduction

Human epidermal growth factor receptor-2 (HER2) status assessment is of pivotal importance for targeted therapy for HER2 positive primary^1–4^ and metastatic^5–7^ breast cancer. The American Society of Clinical Oncology (ASCO) and College of American Pathologists (CAP) guideline for HER2 testing gives recommendations for testing by immunohistochemistry (IHC) and (fluorescence) *in situ* hybridization ((F)ISH). HER2 ISH may be evaluated with a single probe, but more often a dual probe is used^8^. Dual probe ISH includes a probe for the *ERBB2(HER2)* gene and a probe for the centromere of chromosome 17 (CEP17), so copy number changes can be found for both locations.

Loss of one chromosome 17 (leaving only one copy per nucleus) is called monosomy and chromosomal copy number gain (more than 2 copies per nucleus) is called polysomy^9^. Gain of CEP17, which is commonly interpreted as chromosome 17 polysomy, is said to be present in up to 68% of breast carcinomas according to various studies^10,11^. Recently, others have questioned the use of only the centromere region of chromosome 17 as a representative of the whole chromosome, arguing that the presence of true polysomy of chromosome 17 is a rare event^12,13^. Since chromosome 17 polysomy can give difficulties in interpretation of HER2 status assessment results^13–15^, it is vital to know if it is present.

In addition to losses and gains of HER2 and CEP17, also copy number alterations of other parts of chromosome 17 occur. Chromosome 17 includes many genes that are involved in breast carcinogenesis, including tumour-suppressor genes *TP53, BRCA1* and *TOP2A*^11^. The clinical relevance of copy number alterations in these genes is not yet clear.

With the present study we aimed to investigate the presence or absence of chromosome 17 polysomy in relation to CEP17 gains and with respect to HER2 status assessment, by combining *in vitro* assays with clinical validation cases.

## Results

### Breast cancer cell lines

**Figure 1:**
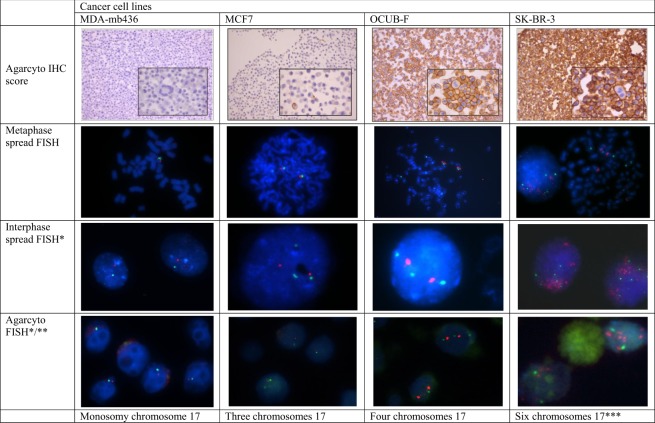
Examples of HER2 IHC of agarcyto slides and HER2*/*CEP17 FISH results on metaphase spreads, interphase nuclei, and agarcyto slides. Due to focusing (*) or cutting artefacts (**), not all of the spots of CEP17 (green) or the *HER2* gene (red) may be visible. ***HER2 gene amplified.

**Figure 2:**
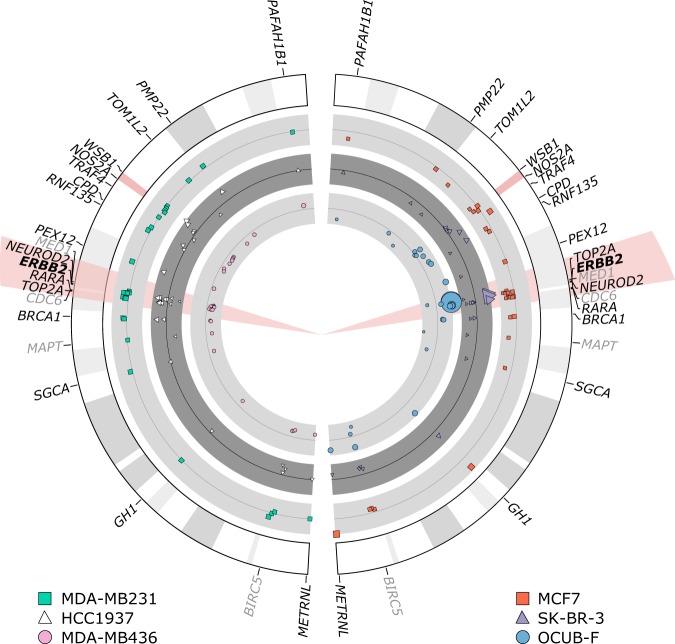
MLPA results of chromosome 17 of all cell lines used. Cell lines MDA-MB231, HCC1937, MCF7, SK-BR-3, OCUB-F and MDA-MB436 are shown. Probemix P004-B1 and P078-B1 (greytone) were used on all cell lines. The *HER2* gene is highlighted in red. The size of the used pictograms corresponds to the reach of the MLPA results (bigger size correlates to higher reach). Results between the probemixes were concordant. The P078-B1 breast tumour probemix also contained probes for genes located on other chromosomes relevant for breast carcinoma and reference genes. In these genes no polysomy or loss or amplification could be detected (not shown in the figure). Negative controls used, were nicely oriented around 1,00 (not shown). The MLPA results with probemix P004-B1 and P078-B1 (greytone) of cell lines MDA-MB231 and HCC1937 show no polysomy or loss or amplification of *HER2*. MCF7 shows partial loss of the *HER2* gene, corresponding to FISH results (Table 1). SK-BR-3 shows amplification of the *HER2* gene corresponding to IHC results and FISH results (Fig. 1). OCUB-F shows amplification of the *HER2* gene corresponding to the IHC and FISH results (Table 1). MDA-MB436 shows partial loss of all tested genes on chromosome 17, concordant with monosomy, as was seen with FISH (Fig. 1).

The analyzed six breast cancer cell lines showed variation in HER2 status (Table 1, Figs 1 and 2): two were amplified and four cell lines were not. There was perfect correlation of the HER2 status when comparing MLPA, IHC, FISH on cells and on metaphase spreads in all cell lines.

**Table 1:** Results of the breast cancer cell lines.

Cell line	HER2 IHC Agarcyto*	HER2 FISH Metaphase spreads (ratio)**	HER2 FISH Agarcyto (ratio)**	HER2 MLPA (ratio)***	CEP17 FISH number of signalsMetaphase spreads	CEP17 FISH number of signals Agarcyto	Copy number chromosome 17 with MLPA	DNA ploidy status
MDA-MB231	0	1.19	0.88	1.23	4	4	2	Polyploid
MDA-MB436	0	1.00	1.00	0.71	1	1	1	Hypoploid
MCF7	1+	0.69	0.85	0.63****	3	3	2	Hyperploid
HCC1937	2+	1.06	1.01	1.21	6	6	2	Hyperploid
OCUB-F	3+	≫2	≫2	7.78	4	4	2	Hyperploid
SK-BR-3	3+	≫2	≫2	6.11	6	5	2	Hyperploid

*Scoring of 0, 1+, 2+ or 3+, **Ratio of spots for the *HER2* gene and CEP17; mean value of 2 independent estimations; ***Ratio as mean value of both MLPA kits used; ****Partial loss of the *HER2* gene.

**Figure 3:**
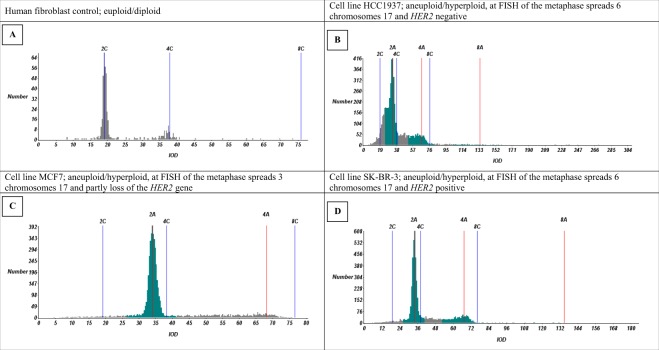
Cell line examples of DNA histograms of agarcyto material. IOD: integrated optical density.

Gain of CEP17 was detected in five of six cell lines with FISH, in both the metaphase spreads and interphase cell nuclei. However, not one cell line showed isolated polysomy of chromosome 17 in the MLPA test (Fig. 2). Especially in metaphase spreads, copy number gain of the whole chromosome 17, and not only of the centromeric region could be appreciated (Fig. 1). Copy number gain of chromosome 17 correlated with a tetraploid or aneuploid pattern with gains in the DNA ploidy assessment in five of six cell lines (three DNA histograms are shown in Fig. 3. These data show that copy number gain of CEP17 is not due to isolated chromosome 17 polysomy, but is a result of aneuploidy with gain of multiple chromosomes.

### Breast cancer cases

Next, we set out to assess whether our findings in the cell lines could be confirmed in 97 breast cancer samples. The patient and tumour characteristics are summarized in Table 2. Based on the centromere probe for chromosome 17, 49 tumours (46.7%) showed copy number gain with 3 or more CEP17 signals (Table 2); 41(91.1%) of these showed aneuploid gains (including tetraploidy) and 4 (8.9%) showed diploidy. As such, finding more than 2 CEP17 signals strongly correlated with aneuploid gains (91.1%, p < 0.001). Pearson’s correlation between the number of CEP17 signals and DNA ploidy status is 0.579, with a sensitivity of 70.7%, a specificity of 89.7% and a positive and negative predictive value of 91.1% and 67.3%, respectively.

**Table 2:** Patient and tumour characteristics.

Patient and tumour characteristics	HER2 +	HER2 −	Total (%)
Age at diagnosis [mean, year (range)]	55.6 (30–78)	57.9 (31–94)	57.2 (30–94)
Origin* (N (%)):			
Primary breast cancer	21 (20.8)	73 (72.2)	94 (96.9)
Breast cancer metastasis	1 (33.3)	2 (66.7)	3 (3.1)
Tumour type* (N (%)):			
Invasive ductal carcinoma	21 (26.6)	58 (73.4)	79 (81.4)
Invasive lobular carcinoma	1 (7.1)	13 (92.9)	14 (14.4)
Apocrine carcinoma	0 (0)	1 (100)	1 (1.0)
Micropapillary invasive carcinoma	0 (0)	1 (100)	1 (1.0)
Mucinous carcinoma	0 (0)	1 (100)	1 (1.0)
Tubular carcinoma	0 (0)	1 (100)	1 (1.0)
Tumour size (in cm)** (N (%)):			
0.5–2.0	9 (22.5)	31 (77.5)	40 (42.1)
2.1–5.0	11 (23.4)	36 (76.6)	47 (48.5)
>5.0	2 (25.0)	6 (75.0)	8 (8.3)
Tumour grade* (N (%)):			
I	0 (0)	12 (100)	12 (12.4)
II	6 (16.7)	30 (83.3)	36 (37.1)
III	16 (34.0)	31 (66.0)	47 (48.5)
Not estimated	0 (0)	2 (100)	2 (2.1)
Oestrogen receptor* (N (%)):			
Positive	16 (19.8)	65 (80.2)	81 (83.5)
Negative	6 (37.5)	10 (62.5)	16 (16.5)
Progesterone receptor*** (N (%)):			
Positive	8 (13.3)	52 (86.7)	60 (61.9)
Negative	14 (38.9)	22 (61.1)	36 (37.1)
Lymph node status**** (N (%)):			
Not applicable	1(33.3)	2 (66.7)	3 (3.2)
Positive	15 (35.7)	27 (64.3)	42 (44.2)
Negative	5 (10.0)	45 (90.0)	50 (52.6)
HER2 status based on HER2/CEP17 FISH (N (%)):			
Positive			22 (22.7)
Negative			75 (77.3)
Partial loss of the *HER2* gene			9 (12.0)
Number of chromosome 17 based on HER2/CEP17 FISH* (N (%)):			
1	2 (15.4)	11 (84.6)	13 (13.4)
2	9 (23.1)	30 (76.9)	39 (40.2)
≥3	11 (24.4)	34 (75.6)	45 (46.4)

*For total of 97 tumours. N: number of cases. **For total of 95 tumours. For two samples the tumour size was unknown/not applicable. ***For total of 96 tumours. Progesterone receptor status was not determined on 1 tumour sample. ****For total of 95 patients.

In accordance to our cancer cell line results, copy number gain of CEP17 is associated with DNA aneuploidy of the tumour with gain of multiple chromosomes. DNA ploidy assessment of the 97 breast tumours analyzed is shown in Table 3. DNA aneuploidy with gains was correlated with p53 mutant protein expression (p = 0,006), but not with DFS or DSS (p = 0.838 and 0.742, respectively) in our population.

**Table 3:** DNA ploidy assessment of 97 breast tumours analyzed.

Characteristics	DNA ploidy status		
	Aneuploid with loss N (%)	Euploid N (%)	Aneuploid with gain* N (%)
HER2 status based on HER2/CEP17 FISH:			
Positive (N = 22)	0 (0)	5 (22.7)	17 (77.3)
Negative (N = 75)	1 (1.3)**	33 (44.0)	41 (54.7)
Partial loss of HER2 (N = 9)	0 (0)	4 (44.4)	5 (55.6)
Number of chromosome 17 based on HER2/CEP17 FISH:			
1	1 (7.7)	9 (69.2)	3 (23.1)
2	0 (0)	25 (64.1)	14 (35.9)
≥3	0 (0)	4 (8.9)	41 (91.1)
p53 protein expression			
Wildtype	1 (1,5)	32 (48,5)	33 (50,0)
Mutant	0 (0)	6 (19,4)	25 (80,6)
Overexpression	0 (0)	5 (21,7)	18 (78,3)
Completely negative	0 (0)	1 (12,5)	7 (87,5)

N: number of cases. *Including tetraploidy. **Probably aneuploid with loss, although morphologically it was not possible to differentiate tumour nuclei from normal nuclei.

**Figure 4:**
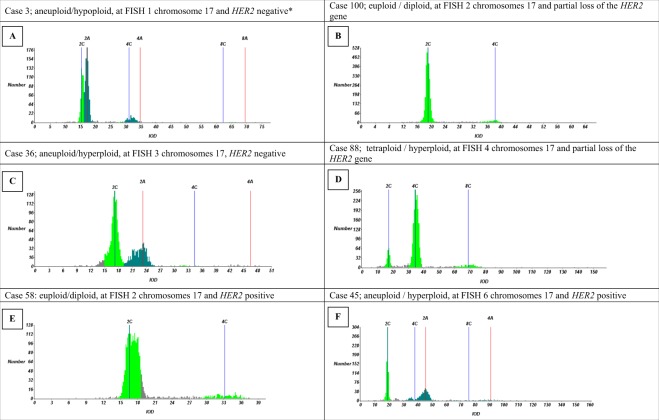
Breast cancer examples of DNA histograms. IOD: integrated optical density. *Probably hypoploidy although morphologically it was not possible to differentiate tumour nuclei from normal nuclei.

Twenty two (22.7%) tumours showed *HER2* gene amplification, 75 (77.3%) showed no amplification, of which nine (8.6%) showed a partial loss of the *HER2* gene using FISH. In the HER2 negative group, 1 case (1.3%) was aneuploid with losses (Fig. 4a), 33 (44.0%) were diploid (Fig. 4b), and 41 (54.7%) aneuploid with gains (Fig. 4c). Of the tumours with a partial loss of the *HER2* gene with the FISH test, 4 (44.4%) were diploid (Fig. 4b) and 5 (55.6%) aneuploid with gains (Fig. 4d). Five (22.7%) of 22 HER2 positive cases were diploid (Fig. 4e) and 17 (77.3%) showed an aneuploid DNA pattern with gains (Fig. 4f).

## Discussion

CEP17 copy number gain can lead to difficulties in the interpretation of HER2 status, but it is still disputed whether this represents true chromosome 17 polysomy. Within the present study, we investigated the presence or absence of chromosome 17 polysomy in relation to CEP17 gains and HER2 status assessment.

Our *in vitro* experiments showed that CEP17 gains are actually gains of the whole chromosome and have a strong correlation with aneuploidy with gain of multiple chromosomes. Isolated chromosome 17 polysomy was not present in any of our cell lines. In our clinical study, over 90% of the patients with CEP17 copy number gain showed aneuploidy with gains. Our results of the cancer cell lines have furthermore shown excellent concordance for HER2 status between IHC, FISH and MLPA, with comparable or better concordance than in previous studies^13,16^.

(F)ISH of CEP17 has generally been used as a surrogate for chromosome 17 copy number assessment in the absence of genetic analysis. Commonly, a mean CEP17 ≥ 3 is adopted as a threshold for polysomy of chromosome 17. As such, the reported prevalence of chromosome 17 polysomy in various studies ranges from 3–68%^10,11^. More recently however, studies using array based techniques^12,14,17^, MLPA^13^ and extended FISH probe panels for chromosome 17^16,18–20^ all show that true polysomy, defined as copy number gain of (almost) all the genes on the chromosome, is a rare event, occurring in <1% of breast cancer cases. Indeed, in the present study we have shown that CEP17 gains in 5 of 6 breast cancer cell lines was not associated with true, isolated polysomy of chromosome 17 in any of these cases. We did show aneuploidy with chromosomal copy number gain in all cell lines with CEP17 gain. So, there seems to be a gain of multiple chromosomes and not only chromosome 17 polysomy in tumours with CEP17 gains. Due to lack of ploidy assessment and metaphase spread assessment in previously conducted studies, this feature has not yet been revealed. Indeed, the methods used so far, MLPA, array-based comparative genomic hybridization (aCGH) and FISH for chromosome 17 with extended probe panels, are not optimally suited to detect polyploidy.

In the past, several studies have hinted at DNA aneuploidy in relation to CEP17 copy number gains, based on the association of copy number increases or “polysomy” of other chromosomes in association with chromosome 17 “polysomy” using a variety of techniques^18,21–23^.

Aneuploidy, which is the presence of an abnormal number of chromosomes in a cell^9^, is the most common characteristic of human solid tumours and is said to contribute to, or even drive tumour development. It results from aberrant mitotic divisions, which can be caused by numerous factors. These include defects in duplication, maturation or segregation of centrosomes, defects in spindle attachment and chromosome cohesion, previous cytokinesis defects and impairment of the mitotic checkpoint response^24^. In invasive breast cancer, approximately 80% of tumours show abnormalities in the structure and/or number of centrosomes, which shows a positive correlation with aneuploidy^25^. Centrosome defects are linked to chromosome missegregation during mitosis, but also to initiating micronucleus formation through merothelic chromosome attachments, chromosome breakage at centromeres, and DNA damage on miss attached chromosomes^26^. p53 status, a centrosome located protein, is also linked to aneuploidy and inactivation of this tumor suppressor can be an underlying genetic cause of centrosome defects. Indeed, also in our population mutant p53 protein expression correlated with DNA aneuploidy (p = 0,006). On the other hand, others have demonstrated that centrosome abnormalities can lead to p53 inactivation^27,28^. Also Nek2, which is a NIMA (never in mitosis A) related kinase, has a function as a major centrosome regulator. This cell cycle-related protein kinase is overexpressed by all breast cancer subtypes compared to healthy tissue and was found to be important for tumor growth at primary and secondary sites. Nek2 knockdown induced aneuploidy and cell cycle arrest that led to cell death, especially in the triple negative subtype, indicating it as a interesting novel therapeutical target. This might sound counterintuitive, but aneuploidy and aberrant mitosis can also cause chromosomal breakage, resulting in a lethal genetic imbalance that leads to cell death^29^. Another interesting novel therapeutical target linked to centrosome function is KLF14 (Kruppel-like family of transcription factors 14). KLF14, which is often downregulated in human carcinomas, has a function in maintaining the integrity of centrosomes by limiting Plk4 (Polo-like kinase 4) transcription, which is a master regulator of centriole duplication and assembly. Loss of KLF14 leads to centrosome amplification, genome instability and spontaneous tumour formation. On the other hand, enforced expression of KLF14 induces mitotic catastrophe, acting as a tumor suppressor^30^.

Established aneuploidy is a significant predictor for breast cancer progression and is correlated to worse clinical outcome^31^. Not only is there a strong correlation between grade and ploidy status, but DNA ploidy can also subdivide patients with low grade breast carcinoma into different prognostic groups^32^. This finding can be of great clinical value, as it can help identify patients with low grade tumours who might benefit from adjuvant chemotherapy. CEP17 was shown to be a potential marker for aneuploidy, but also other centromere probes could be of interest. As dual probe FISH is already validated and often used in daily clinic practice for HER2 evaluation however, it will be feasible to also report the presence of copy number gains of CEP17 in pathology reports. This can trump subsequent ploidy assessment, which can be used for therapeutic decision-making in the future. Techniques like MammaPrint and Oncotype DX are already used by some as a tool to determine if adjuvant chemotherapy should be added to the treatment of patients with well and moderately differentiated tumours^33^. We propose the exploration for a similar role for the already widely used and relatively cheap dual probe FISH technique in larger cohorts. In our cohort, which was mixed with both primary and metastatic lesions and where patients received different treatments, we did not see a correlation between aneuploid gains and DFS or DSS. Our cohort however, was not selected for this purpose and is as such too heterogeneous, relatively small and has a short follow-up (median of 58 months). Pinto *et al*.^32^ for instance, who found that aneuploidy can identify subsets of patients with poor clinical outcome in grade 1 and grade 2 tumours, included 684 patients with a median follow-up of 134.5 months. CEP17 copy number gain has furthermore also shown to be correlated to response to anthracycline based chemotherapy^34,35^. Considering above mentioned added value of combining a probe for CEP17 and a probe for the *HER2/ERBB2* gene when performing *HER2/ERBB2* gene ISH, we would like to advocate the use of a double probe ISH in favour of a single probe ISH in all breast carcinomas.

Our results show that in a few of our breast tumours, CEP17 copy gain, based on the double probe FISH test, did not correlate with DNA aneuploid gains. There were 4 (8.9%) tumours with ≥ 3 CEP17 FISH signals and with a diploid DNA status. Isolated centromere gains of chromosome 17 might explain why not all CEP17 gains are correlated with aneuploid gain^12,13^. Another explanation is variances in CEP17 signals due to genomic heterogeneity, proliferative activity and sectioning artefacts^11^. HER2 partial loss, as seen in nine of our cases (Table 3), can be due to rearrangement of chromosomes^36^.

It is of relevance to know if CEP17 gains are of importance for HER2 interpretation. The current ASCO/CAP guideline defines tumours as HER2 positive if the HER2/CEP17 ratio is ≥2.0 *or* if there are ≥6 *HER2* genes per nucleus^8^. The addition of the latter was in order to minimize the effect of CEP17 variation on HER2 test results^37^. It is important to note that HER2 gains, both in absolute number and when considering the HER2/CEP17 ratio, are positively correlated with HER2 protein over-expression^38^. Furthermore, previous studies have shown that tumours with CEP17 gains in the absence of *HER2* gene amplification resemble HER2 negative tumours, and do not seem to yield sensitivity for anti-HER2 directed agents^15,39^. Also, CEP17 gain seems not to be associated with clinical outcome in both the HER2 negative and the HER2 positive situation^15,40^.

## Conclusions

This study has shown that copy number gain of CEP17, which is encountered regularly in HER2 testing of breast cancer, is due to gain of chromosome 17, which is a result of aneuploidy of the tumour with gain of multiple chromosomes. As aneuploidy of breast carcinomas is associated with poor clinical outcome, also within low grade tumours, gain of CEP17 (and the associated correlation with aneuploid gain) might be used to select patients with a poor outcome and offer them adjuvant chemotherapy. Additional studies are however warranted to confirm our results and further test this hypothesis.

## Methods

### Cancer cell lines

Six breast cancer cell lines (MDA-MB231, OCUB-F, SK-BR-3, MCF7, MDA-MB436 and HCC1937) were used. All cancer cell lines were obtained from LGC Standards (Teddington, Middlesex, UK), with exception of the OCUB-F cell line, which was from Riken Gene Bank (Tsukuba, Japan). Identity of cell lines was authenticated by the suppliers by comparing growth properties and morphology. Species confirmation was performed by cytochrome oxidase I isoenzyme testing, and short tandem repeat analysis. Prior to this study, SK-BR-3 was known to be HER2 positive, HCC1937 was known to be HER2 negative, while no HER2 status was available for the other cell lines.

### Cell culture

The breast cancer cell lines used were maintained in Dulbecco’s modified Eagle’s medium (DMEM, Thermo Fisher Scientific Inc., Waltham, MA, USA) supplemented with 10% (vol/vol) fetal bovine serum (FBS, Sigma-Aldrich corporation, St. Louis, MO, USA), 20 mM Hepes, 1× nonessential amino acid, 2 mM L-glutamine and 10 U/ml penicillin, 10 μg/ml streptomycin (all from PAA Laboratories, Cölbe, Germany) at 37 °C with 5% CO_2_ as described before^41^. Hanks’ Balanced Salt Solution (HBSS, PAA Laboratories) was used as a washing. Cells were incubated under standard conditions before processing. Metaphase and interphase spreads for subsequent FISH analysis were prepared according to standard procedures^42^.

### Agarcyto cell block preparation

We modified the agarcyto cell block preparation, which was described previously^43^; the first centrifugation was performed for 10 min at 217 × *g* and the second for 10 min at 867 RCF. The pellet was resuspended in 1 ml 2.25% liquid agarose (Agar technical, Agar No 3, Oxoid BV, Badhoevendorp, the Netherlands) at 60 °C. The third centrifugation was 10 min at 867 RCF to concentrate the cells in the agar. The agar cell pellet was then solidified at 4 °C for 30 min. The tissue was embedded in paraffin using an automated tissue processor (Pathos; Milestone, Sorisole (BG), Italy) under standard conditions for surgical biopsies. From the agarcyto block, 4 µm sections were cut and stained with hematoxylin-eosin (H&E) for cytomorphological examination.

### P53 and HER2 IHC and HER2 FISH

P53^44^ and HER2 IHC^45^ and HER2 FISH^46^ were performed and scored as described elsewhere. For the ISH process, custom made probe *ERBB2-HER2/Neu* 17Q12/SE-17 (KB-00007 Kreatech, Leica, Rijswijk, The Netherlands) was used. Scoring of HER2 IHC and FISH was conducted as described by the latest ASCO/CAP guideline for HER2 status assessment^8^.

### Multiplex ligation-dependent probe amplification (MLPA)

DNA was isolated with the QIAamp DNA micro kit (category no. 56304, Qiagen, Venlo, The Netherlands) according to the manufacturer’s instructions, with a few alterations. Step 12 included adding of 30 µl elution AE buffer (Qiagen) on the column and incubation for 1 min at room temperature before centrifugation for 1 min at 6000 × *g*. This was repeated once more, before the isolated DNA was judged for concentration and purity using the Nanodrop Spectrophotometer (NanoDrop Rechnologies Inc., Wilmington, DE, USA).

Next the DNA solution was used in the MLPA analysis according to the manufacturer’s instructions, using the P004-B1 ERBB2 probemix and the P078-B1 breast tumour probemix (both MRC Holland, Amsterdam, The Netherlands; access to precise contents via www.mlpa.com). PCR products were analyzed in a 3730XL DNA Analyzer. Gene copy numbers were analyzed using Genemapper v4.0 software. This software analyzed peak heights of all the genes present in the MLPA kits. These numbers were implemented in an excel template and values were automatically calculated and formed into a copy number graph. A value between 0.7 and 1.3 was defined as normal. A value below 0.7 was defined as a gene lost, a value between 1.3 and 2.0 as low level amplification and values > 2.0 as high level amplification according to the definitions in the software.

### Breast cancer cases

From December 2010 until March 2013 a total of 698 breast carcinomas were tested for HER2 with a double probe FISH test at the department of pathology of the Radboud university medical center (Radboudumc) in Nijmegen, The Netherlands. From this series, a total of 97 tumours were selected for this study, based on the availability of a resection specimen for DNA ploidy assessment, which was dependent on the size of the tumour and quality of DNA. Patients were either treated in hospital Pantein in Boxmeer or in the Radboudumc in Nijmegen. No ethical approval was required according to current Dutch legislation, as we used leftover coded material^47^ and patients were given the opportunity to object to their leftover material to be used in (clinical) research. Following the observation of aneuploidy, survival data were also obtained for all cases. Disease free survival (DFS) was determined from the date of the initial diagnostic biopsy until affirmed recurrence of disease and disease specific survival (DSS) from the date of the initial diagnostic biopsy until death due to breast cancer.

### DNA ploidy measurement of isolated nuclei from thick sections

Feulgen-Schiff stained cytospins containing nuclei isolated from 50 μm thick sections were prepared as described before^48^, and in accordance with the consensus criteria of the European Society for Analytical Cellular Pathology (ESACP)^49^. Briefly, nuclei were isolated using digestion with 2 ml 0.5% (w/v), pepsin pH1.5 (Sigma Aldrich, St. Louis, MO) for 60 min at 37 °C. Five min digestion was used for sections from agarcyto embedded tumour cell cultures. Hydrolysis was performed using 5 N HCL at 25 °C for 60 min. Next, specimens were stained with Schiff’s reagent (Merck, Darmstadt, Germany) at RT for 60 min.

DNA ploidy status was assessed according to the ESACP criteria^49^, using a QPath image cytometry station (Leica Microsystems GmbH, Wetzlar, Germany). The system acquires digital images using a monochromatic CCD camera attached to a Leica DM LB2 microscope using a 40x objective (Leica HCX PL Fluotar, N.A. = 0.75; resulting specimen level pixel size 0.326 × 0.326 μm^2^). Cytospins were measured fully automatically. The system allows definition of criteria that objects have to meet before being included in the final data set. Criteria applied in this study were: area between 50 and 850 pixels and roundness (defined as perimeter^2^/(4 * PI * area)) smaller than 1.25. Based on previous experience, these criteria minimize the amount of debris entering the analysis without rejecting true nuclear objects. DI histograms were manually classified by three independent observers (JvdL, IO-H, DV). In case of interobserver discrepancy, consensus was reached by jointly evaluating cases. Histograms were classified as: tetraploid histogram: >15% of cells in the tetraploid region and 5c exceeding rate >1%; aneuploid histogram: presence of a peak outside the diploid and tetraploid regions, or 2.5% of cells with DNA index exceeding 5c; or diploid histogram: all others.

### Statistical analysis

Data was analyzed using IBM SPSS Statistics for Windows (version 20.0, IBM Corp., Armonk, NY, USA). The statistical correlation between the number of CEP17 signals and ploidy status were assessed using Pearson’s rho and the correlation between p53 status and ploidy status using Pearson’s Chi-Square test of independence. P values less than 0.05 were considered significant. Sensitivity, specificity, positive- and negative predictive values were calculated in order to determine the accuracy of the amount of CEP17 signals in predicting DNA ploidy status assessment. DFS and DSS curves were estimated by the Kaplan-Meier method and compared by means of the log-rank test for all primary tumours.

## Data Availability

The datasets generated and analysed during the current study are available from the corresponding author on reasonable request.
